# Rhodamine 6G/Transition Metal Dichalcogenide Hybrid Nanoscrolls for Enhanced Optoelectronic Performance

**DOI:** 10.3390/molecules29122799

**Published:** 2024-06-12

**Authors:** Huihui Ye, Hailun Tang, Shilong Yu, Yang Yang, Hai Li

**Affiliations:** Key Laboratory of Flexible Electronics (KLOFE), Institute of Advanced Materials (IAM), Nanjing Tech University (NanjingTech), Nanjing 211816, China

**Keywords:** MoS_2_ nanoscrolls, organic photoactive materials, solution treatment, photoelectric properties, type II heterojunction

## Abstract

The low light absorption efficiency has seriously hindered the application of two-dimensional transition metal dichalcogenide (TMDC) nanosheets in the field of optoelectronic devices. Various approaches have been used to improve the performance of TMDC nanosheets. Preparation of one-dimensional TMDC nanoscrolls in combination with photoactive materials has been a promising method to improve their properties recently. In this work, we report a facile method to enhance the optoelectronic performance of TMDC nanoscrolls by wrapping the photoactive organic dye rhodamine (R6G) into them. After R6G molecules were deposited on monolayer TMDC nanosheets by the solution method, the R6G/MoS_2_ nanoscrolls with lengths up to hundreds of microns were prepared in a short time by dropping a mixture of ammonia and ethanol solution on the R6G/MoS_2_ nanosheets. The as-obtained R6G/MoS_2_ nanoscrolls were well characterized by optical microscopy, atomic force microscopy, Raman spectroscopy, and transmission electron microscopy to prove the encapsulation of R6G. There are multiple type II heterojunction interfaces in the R6G/MoS_2_ nanoscrolls, which can promote the generation of photo-induced carriers and the following electron–hole separation. The separated electrons were transported rapidly along the axial direction of the R6G/MoS_2_ nanoscrolls, which greatly improves the efficiency of light absorption and photoresponse. Under the irradiation of an incident 405 nm laser, the photoresponsivity, carrier mobility, external quantum efficiency, and detectivity of R6G/MoS_2_ nanoscrolls were enhanced to 66.07 A/W, 132.93 cm^2^V^−1^s^−1^, 20,261%, and 1.25 × 10^12^ cm·Hz^1/2^W^−1^, which are four orders of magnitude higher than those of monolayer MoS_2_ nanosheets. Our work indicates that the R6G/TMDC hybrid nanoscrolls could be promising materials for high-performance optoelectronic devices.

## 1. Introduction

Over the past decade, two-dimensional (2D) transition metal dichalcogenides (TMDC) materials have been widely used in photodetectors [1,2,3,4,5], solar cells [6,7,8,9,10], and catalytic hydrogen evolution [11,12,13,14,15,16,17,18], due to their excellent photoelectric properties. However, the low visible light absorption of atomically thin TMDC nanosheets restricts their practical application in developing high-quality photodetectors [19]. In order to solve this challenge, various methods have been proposed to enhance the light absorption and improve the performance of TMDC nanosheet-based photodetectors, including integrating photosensitive materials [20,21], doping with chemicals [22,23], and forming heterojunction [24,25,26,27], etc. Among them, the involvement of organic dye molecules is considered one of the most simple and effective methods. As a kind of photoactive material, organic dyes have been widely used in the fields of sensitized solar cells [28,29] and optoelectronic devices [21,30,31], due to their easy access and unique optical properties [31,32]. Among the photoactive organic dyes, rhodamine 6G (R6G) is a promising material with good photostability, high quantum yield, and excellent light absorption ability [20,21]. When R6G is irradiated by light, the electron energy levels can form an extended π-electron cloud as they pass within the molecule, making the R6G molecule more sensitive to light absorption and photoresponse. By drop-casting R6G molecules on a monolayer MoS_2_ nanosheet, the photocurrent was enhanced by an order of magnitude. Thus, the photoresponsivity and detectivity were improved by 10 times in a broad wavelength range (λ = 400–800 nm) [31]. Similar performance enhancement was also found in an MoS_2_ nanosheet-based photodetector by dropping organic dyes of R6G, methyl orange, and methylene blue, which was attributed to the photoinduced charge transfer from the organic dyes to the MoS_2_ nanosheet as well as the optimized light absorption range [21]. When monolayer R6G film was sandwiched between two graphene layers to form the graphene-R6G-graphene (G-R-G) heterostructure, the Raman signal of the G-R-G heterostructure was about 7 times better than that of the R6G-graphene (R-G), arising from the photoinduced Dirac voltage change. In addition, the sandwiched R6G film could act as a photoactive layer, resulting in photoresponsivity enhanced by 40 times higher than that of the R-G photodetector [32]. The great optoelectronic performance improvement could be assigned to the efficient charge transfer between the organic dye R6G and graphene because of the weak π-π interactions in graphene layers. Similarly, R6G molecules were also trapped into mechanically exfoliated BP/MoS_2_ nanosheets to form BP/R6G/MoS_2_ type Ⅰ heterojunction. Due to the existence of a built-in electric field at the BP/MoS_2_ interface, ultrafast separation of the photogenerated carriers in R6G molecules could be observed, which greatly enhances the photoresponsivity and response speed of the BP/MoS_2_ p-n heterojunction [20].

Recently, it has been reported that the optoelectronic performance of 2D TMDC nanosheets can be greatly improved by transforming into one-dimensional (1D) spiral nanoscrolls [33,34,35,36]. The as-formed nanoscrolls not only retain the excellent properties of TMDC nanosheets but also exhibit increased cross-sectional area for light absorption [37]. At the same time, the spirally scrolled structure restricts the transportation of electrons along the long axis, which also greatly improves the generation of photoinduced carriers [19,38,39]. In addition, the adjustable layer spacing and open ends of nanoscrolls facilitate the encapsulation of various functional materials. For instance, after Ag nanoparticles were encapsulated into MoS_2_ nanoscrolls, the photosensitivity of MoS_2_ nanoscrolls was improved by 20 times [19]. When carbon quantum dots were encapsulated into MoS_2_ nanoscrolls, the photoluminescence of MoS_2_ nanoscrolls was also improved by 830 times [40]. Two orders of magnitude increases in photosensitivities have been observed by encapsulating PbI_2_ nanoparticles into MoS_2_ nanoscrolls [35]. Moreover, by encapsulating BaTiO_3_ nanoparticles in MoS_2_ nanoscrolls [38], the photoresponsivity and external quantum efficiency of MoS_2_ nanoscrolls increased by almost two orders of magnitude. These results indicate that combining nanoscrolls with photoactive materials is a promising way to enhance the optoelectronic performance of TMDC nanoscrolls. Since the organic dye R6G has exhibited great enhancement on the optoelectronic performance of 2D TMDC nanosheets, it is interesting to encapsulate R6G into TMDC nanoscrolls to further improve the optoelectronic performance.

In this work, the monolayer TMDC nanosheets (MoS_2_ and WS_2_) were first grown by chemical vapor deposition (CVD). Then they were immersed in the R6G solution (Macklin, Shanghai, China) at different concentrations for various periods of time. After that, the R6G molecules were uniformly deposited on TMDC nanosheets with varied amounts by controlling the concentration and immersion time to obtain the R6G/TMDC nanosheets, such as R6G/MoS_2_ and R6G/WS_2_. A mixture of ethanol and ammonia (Yonghua Chemical, Changshu, China) was added dropwise onto the R6G/TMDC nanosheets under heated conditions to prepare the R6G/MoS_2_ and R6G/WS_2_ nanoscrolls with lengths of hundreds of micrometers in a few seconds. The as-prepared R6G/MoS_2_ nanosheets and R6G/MoS_2_ nanoscrolls were characterized using optical microscopy (OM), Raman spectroscopy, atomic force microscopy (AFM), and transmission electron microscopy (TEM). The amount of R6G deposited on the MoS_2_ nanosheets increased with the increasing concentration of R6G and immersion time. Compared to MoS_2_ nanosheets and MoS_2_ nanoscrolls, the as-prepared R6G/MoS_2_ nanoscrolls exhibited excellent responsivity (R), carrier mobility (μ), external quantum efficiency (EQE), and detectivity (D*), which are improved by several orders of magnitude. The excellent optoelectronic performance of R6G/MoS_2_ nanoscrolls could be attributed to the fast charge transfer between the R6G and MoS_2_ interfaces as well as the spirally scrolled structure.

## 2. Results

**Figure 1:**
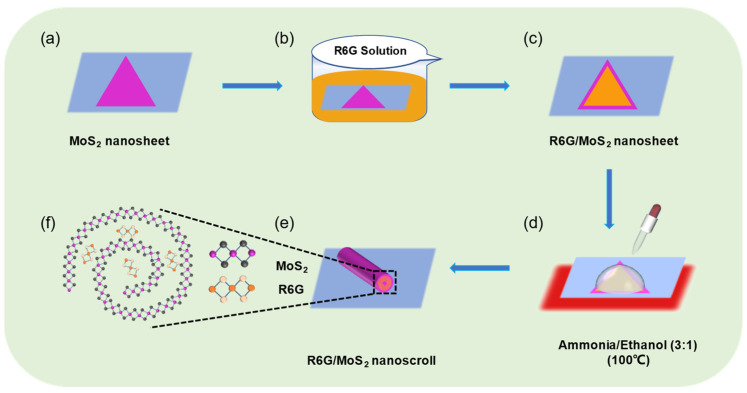
The schematic preparation of R6G/MoS_2_ nanosheet and nanoscroll. (**a**) Monolayer MoS_2_ nanosheet was grown on SiO_2_/Si substrates by CVD method. (**b**,**c**) Monolayer MoS_2_ nanosheet was (**b**) immersed in R6G solution for 10 min to obtain (**c**) R6G/MoS_2_ nanosheet. (**d**) A mixed solution of ammonia and ethanol (3:1) was dropped on the R6G/MoS_2_ nanosheet at 100 °C. (**e**) The as-obtained R6G/MoS_2_ nanoscroll. (**f**) The atomic structure of R6G/MoS_2_ nanoscroll.

The preparation process of R6G/MoS_2_ nanosheets and R6G/MoS_2_ nanoscrolls is illustrated in Figure 1. Firstly, monolayer MoS_2_ nanosheets were grown on SiO_2_/Si substrates by the CVD method, as shown in Figure 1a. Secondly, the as-grown MoS_2_ nanosheets were immersed in the R6G solution with a concentration of 5.0 mM for 10 min to obtain the R6G/MoS_2_ nanosheets (Figure 1b,c). The as-prepared R6G/MoS_2_ nanosheets were washed by deionized water several times to wash off the R6G molecules attached to the SiO_2_/Si substrate and dried with N_2_. Finally, a droplet of an ethanol/ammonia mixture (3:1) was dropped on the R6G/MoS_2_ nanosheets at 100 °C to induce the formation of R6G/MoS_2_ nanoscrolls in a short time (Figure 1d). In this process, the SiO_2_ layer was etched by an alkali solution that infiltrated the interface between the R6G/MoS_2_ nanosheets and SiO_2_/Si substrate. Therefore, the adhesion force balance between R6G/MoS_2_ nanosheets and SiO_2_/Si substrate was broken, making the edge of the nanosheets roll up until they form nanoscrolls (Figure 1e,f).

**Figure 2:**
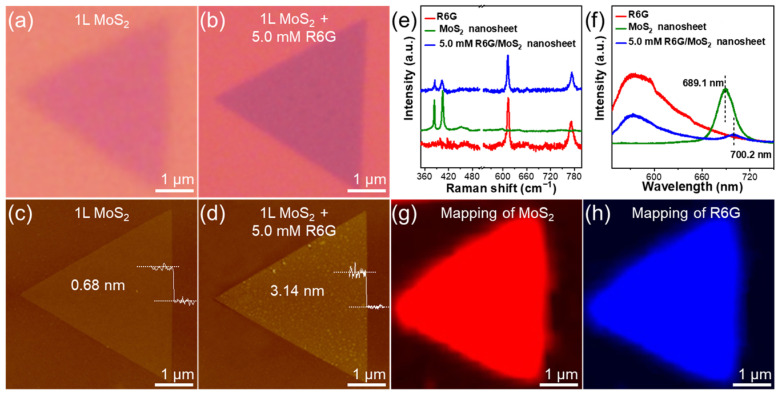
(**a**) The optical and (**c**) AFM images of MoS_2_ nanosheet. (**b**) The optical and (**d**) AFM images of MoS_2_ nanosheet immersed in 5.0 mM R6G solution for 10 min. (**e**) Raman and (**f**) PL spectra of MoS_2_ nanosheet, R6G, and R6G/MoS_2_ nanosheet. (**g**,**h**) Raman mapping images of (**g**) MoS_2_ in the range of 350–420 cm^−1^ and (**h**) R6G in the range of 600–800 cm^−1^ in R6G/MoS_2_ nanosheet.

Figure 2a,b show the optical images before and after the MoS_2_ nanosheet was immersed in the R6G solution for 10 min. It can be observed that the color of the R6G/MoS_2_ nanosheet becomes darker than the pristine one, indicating the successful deposition of R6G. AFM characterization also reveals the height change of the MoS_2_ nanosheet before and after R6G modification (Figure 2c,d). The thickness of MoS_2_ nanosheet is 0.68 nm (Figure 2c), confirming its monolayer structure [35]. After immersing in a 5.0 mM R6G solution for 10 min, the thickness of the nanosheet increased to 3.14 nm, as shown in Figure 2d. It demonstrates that R6G molecules have been successfully adsorbed on the MoS_2_ nanosheet. Raman spectroscopy and mapping can also be used to prove the presence of R6G on MoS_2_ nanosheets. As shown in Figure 2e, there are two strong peaks located at 385 and 403 cm^−1^ in the Raman spectrum of MoS_2_ nanosheet, respectively, which are assigned to the $E_{2g}^{1}$ and $A_{1g}$ peaks. The peak position difference between the $E_{2g}^{1}$ and $A_{1g}$ peaks is 18 cm^−1^, verifying the existence of monolayer MoS_2_. The characteristic Raman peaks of R6G are located at 613 and 772 cm^−1^, which can be clearly observed in Figure 2e. These four characteristic peaks of R6G and monolayer MoS_2_ appear in the Raman spectrum of the R6G/MoS_2_ nanosheet, indicating that R6G molecules have been successfully modified on the MoS_2_ nanosheet. The monolayer MoS_2_ displays a strong photoluminescence (PL) peak at 689.1 nm, which is red-shifted to 700.2 nm after modification of R6G, as shown in Figure 2f. The peak intensity decreases dramatically, which may be attributed to the formation of a type II heterogeneous interface between R6G and MoS_2_. Figure 2g,h demonstrates the Raman mapping images of the R6G/MoS_2_ nanosheet in the range of 350–420 cm^−1^ and 600–800 cm^−1^, respectively. As shown in Figure 2h, the R6G molecules are uniformly distributed on the MoS_2_ nanosheet.

**Figure 3:**
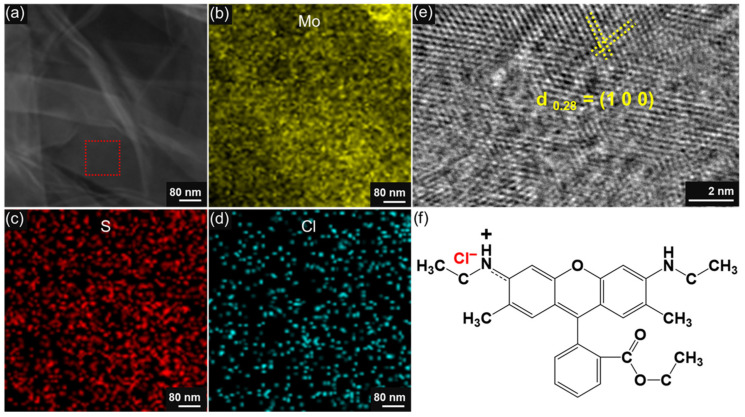
(**a**) TEM image of R6G/MoS_2_ nanosheet. (**b**–**d**) The energy dispersive spectroscopy mapping analysis on the distribution of (**b**) Mo, (**c**) S, and (**d**) Cl elements. (**e**) Magnified HR-TEM image of R6G/MoS_2_ nanosheet marked by dashed red box shown in (**a**). (**f**) The molecular formula of R6G.

To further demonstrate the uniform presence of R6G on the MoS_2_ nanosheet, we also performed TEM characterization (Figure 3). Figure 3a shows the TEM image of R6G/MoS_2_ nanosheets. Since R6G is a small organic molecule, it is difficult to image it even with high-resolution TEM (HR-TEM). As shown in the structural formula of R6G (Figure 3f), there is a characteristic Cl atom in one R6G molecule, which can be used to indicate the presence of R6G. It is well known that energy dispersive spectroscopy (EDS) is a powerful tool to demonstrate the elemental distribution of materials. Figure 3b–d show the EDS mapping images of the R6G/MoS_2_ nanosheet marked by the red dashed box shown in Figure 3a. It can be seen that the Mo, S, and Cl elements are uniformly distributed, indicating that R6G was successfully modified on the MoS_2_ nanosheets. Figure 3e shows the HR-TEM image of the R6G/MoS_2_ nanosheet, and the (1 0 0) crystalline plane of MoS_2_ proves the high quality of MoS_2_ nanosheets.

**Figure 4:**
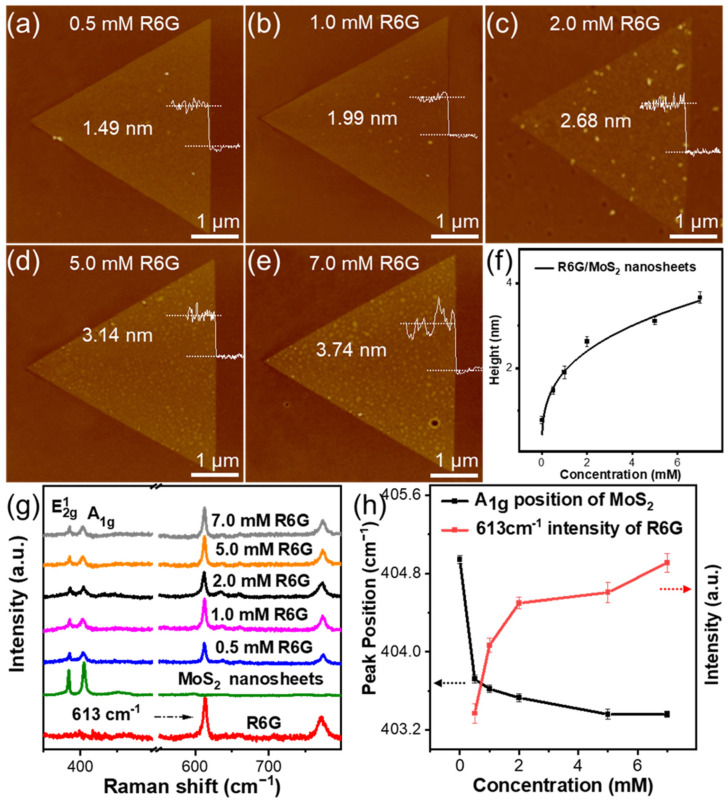
AFM height images of R6G/MoS_2_ nanosheet by immersing in (**a**) 0.5 mM, (**b**) 1.0 mM, (**c**) 2.0 mM, (**d**) 5.0 mM and (**e**) 7.0 mM of R6G solution. (**f**) The height of R6G/MoS_2_ nanosheet as a function of concentration of R6G solution. (**g**) The Raman spectra of MoS_2_ nanosheet, R6G, and R6G/MoS_2_ nanosheet immersed in R6G solution with various concentration. (**h**) Raman shift of the A_1g_ peak of MoS_2_ in R6G/MoS_2_ nanosheet and the intensity of the characteristic peak of R6G (613 cm^−1^) as a function of concentration of R6G solution.

In order to investigate the influence of concentration on the deposition of R6G on MoS_2_ nanosheets, the CVD-grown monolayer MoS_2_ nanosheets were immersed into R6G solutions with concentrations of 0.5, 1.0, 2.0, 5.0, and 7.0 mM, respectively. AFM was employed to monitor the deposition amount of R6G by measuring the height change after modification. As shown in Figure 4a, the thickness of the MoS_2_ nanosheet increases from 0.68 nm to 1.49 nm after immersion in a 0.5 mM R6G solution for 10 min (referred to as the 0.5 mM R6G/MoS_2_ nanosheet), indicating the R6G molecules have been deposited on MoS_2_. The root-mean-square (rms) surface roughness of a pristine MoS_2_ nanosheet is 0.14 nm. While the rms surface roughness of the 0.5 mM R6G/MoS_2_ nanosheet slightly increases to 0.19 nm, implying the uniform deposition of R6G. With the concentration of R6G increasing from 1.0 mM to 7.0 mM, the thickness of MoS_2_ nanosheets also gradually increased from 1.99 nm to 3.74 nm, as shown in Figure 4b–e. Figure 4f also demonstrates the height increase in R6G/MoS_2_ nanosheets with the increasing concentration of R6G. Meanwhile, the $A_{1g}$ peak of MoS_2_ nanosheets was red-shifted from 404.94 cm^−1^ to 403.36 cm^−1^ (Figure 4g,h), implying the influence of an increased amount of R6G. In addition, the intensity of the characteristic peak of R6G at 613 cm^−1^ increased while the intensity of $E_{2g}^{1}$ and $A_{1g}$ peaks decreased, confirming the increased amount of R6G on the MoS_2_ nanosheet (Figure 4h). The effect of immersion time in the R6G solution on the height change of MoS_2_ nanosheets was also explored (Figure S1 in Supporting Information (SI)). As the immersion time increased from 1 min to 30 min, the height of the 5.0 mM R6G/MoS_2_ nanosheets gradually increased from 1.79 nm to 4.02 nm. While heterogeneous R6G aggregation was observed when MoS_2_ nanosheets were immersed in a 5.0 mM R6G solution for 60 min. In order to balance the deposition amount of R6G on MoS_2_ nanosheets and the following preparation of nanoscrolls, the optimal condition for obtaining R6G/MoS_2_ nanosheets was conducted by immersing in a 5.0 mM R6G solution for 10 min.

**Figure 5:**
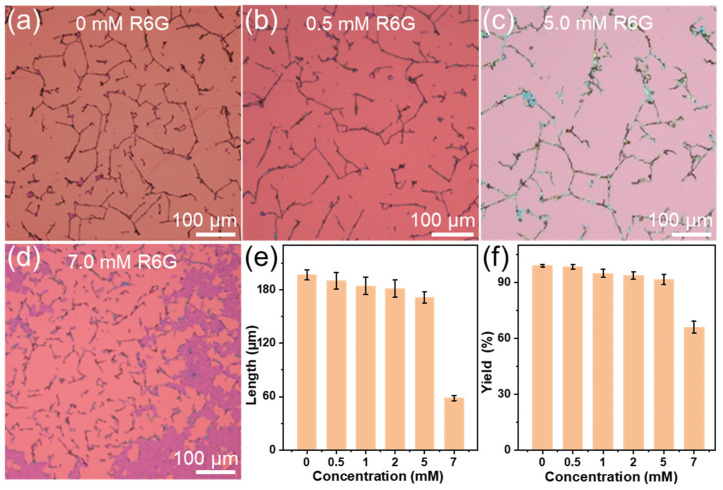
The OM images of (**a**) MoS_2_ nanoscroll and (**b**) 0.5 mM, (**c**) 5.0 mM, and (**d**) 7.0 mM R6G/MoS_2_ nanoscrolls. (**e**,**f**) The (**e**) length and (**f**) yield of R6G/MoS_2_ nanoscroll as a function of concentration of R6G solution.

It has been reported that the monolayer TMDC nanosheets could be transformed into 1D nanoscrolls by using solutions such as ethanol, water, and NaHCO_3_ solution [19,36]. However, the R6G molecules deposited on MoS_2_ nanosheets increase the thickness of the nanosheet and thus decrease its flexibility. As a result, it is difficult to roll up R6G/MoS_2_ nanosheets directly by using these solutions (Figure S2 in SI). In order to roll up the R6G/MoS_2_ nanosheets, a mixture of ammonia and ethanol was used to prepare R6G/MoS_2_ nanoscrolls at 100 °C. It was found that the optimized volume ratio of ammonia to ethanol is 3:1 (Figure S3 in SI). The effect of R6G solution concentration on the preparation of R6G/MoS_2_ nanoscrolls was investigated (Figure 5a–d). For monolayer MoS_2_, almost all MoS_2_ nanosheets were transformed into 1D nanoscrolls with a length of 196.5 ± 5.6 μm (Figure 5a,e), and the yield of nanoscrolls is 99.02 ± 0.58%. When a mixture of ammonia and ethanol was dropped on 0.5 mM R6G/MoS_2_ nanosheets at 100 °C, nanoscrolls with a length of 190.2 ± 9.2 μm were obtained (Figure 5b,e), and the yield was similar to that of a pristine monolayer MoS_2_ nanosheet. As the concentration of R6G increased from 1.0 mM to 5.0 mM, the yield of nanoscrolls decreased from 94.98 ± 2.25% to 91.68 ± 2.75% (Figure 5c,f), and the length decreased from 184.5 ± 10.0 μm to 171.4 ± 6.4 μm (Figure 5c,e). As the concentration of R6G further increased to 7.0 mM (Figure 5d), only a small part of the nanosheets was transformed into nanoscrolls, with the yield decreasing to 66.04 ± 3.1% (Figure 5f) and the length decreasing to 58.29 ± 2.93 μm (Figure 5e). Considering the length and yield of R6G/MoS_2_ nanoscrolls, a 5.0 mM R6G solution was selected for the subsequent preparation of R6G/MoS_2_ nanoscrolls (referred to as 5.0 mM R6G/MoS_2_ nanoscrolls) and optoelectronic performance measurement.

**Figure 6:**
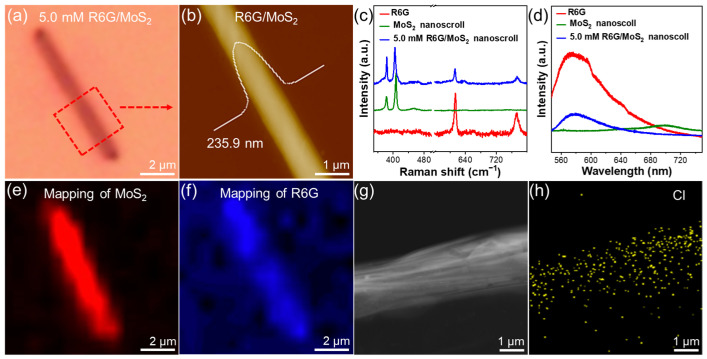
(**a**) OM and (**b**) AFM images of R6G/MoS_2_ nanoscroll. (**c**,**d**) Raman and photoluminescence spectra of R6G/MoS_2_ nanoscroll, MoS_2_ nanoscroll and R6G. (**e**,**f**) Raman mapping images of (**e**) MoS_2_ in the range of 350–420 cm^−1^ and (**f**) R6G in the range of 600–800 cm^−1^ in R6G/MoS_2_ nanoscroll. (**g**) TEM image of R6G/MoS_2_ nanoscroll. (**h**) EDS image of Cl element in R6G/MoS_2_ nanoscroll.

Figure 6a,b show the OM and AFM images of 5.0 mM R6G/MoS_2_ nanoscroll. The R6G/MoS_2_ nanoscroll shows a height of 235.9 nm and a diameter of around 1 μm (Figure 6b). HR-TEM characterization indicates that the R6G/MoS_2_ nanoscroll has a layer spacing of 0.59 nm (Figure S4 in SI), proving the closely packed structure of the R6G/MoS_2_ nanoscroll. Compared to the PL peak of the MoS_2_ nanoscroll located at 702 nm, the R6G/MoS_2_ nanoscroll shows a red-shifted PL peak at 712 nm (Figure 6d). The phenomenon is similar to that of the red-shifted PL peak of R6G/MoS_2_ nanosheets (Figure 2f). Figure 6c shows the Raman spectra of R6G/MoS_2_ nanoscroll, MoS_2_ nanoscroll, and R6G. The $A_{1g}$ peak of MoS_2_ in the R6G/MoS_2_ nanoscroll is red-shifted compared to that of the MoS_2_ nanoscroll, which is also similar to the red-shifted $A_{1g}$ peak of the R6G/MoS_2_ nanosheets shown in Figure 2e. These phenomena indicate the effect of wrapped R6G in MoS_2_ nanoscrolls. In addition, the peaks at 613 cm^−1^ and 772 cm^−1^ in the R6G/MoS_2_ nanoscrolls further confirm the presence of R6G. Figure 6e,f shows the Raman mapping images of R6G/MoS_2_ nanoscrolls in the range of 350–420 cm^−1^ for MoS_2_ (Figure 6e) and 600–800 cm^−1^ for R6G (Figure 6f), demonstrating that R6G is uniformly distributed in MoS_2_ nanoscrolls. The TEM characterization and EDS mapping analysis were also used to confirm the existence of R6G in the R6G/MoS_2_ nanoscrolls (Figure S5 in SI). As presented in Figure 6g,h and Figure S5 in SI, the even distribution of S, Mo, and Cl elements across the R6G/MoS_2_ nanoscroll demonstrates the successful encapsulation of R6G in MoS_2_ nanoscroll.

**Figure 7:**
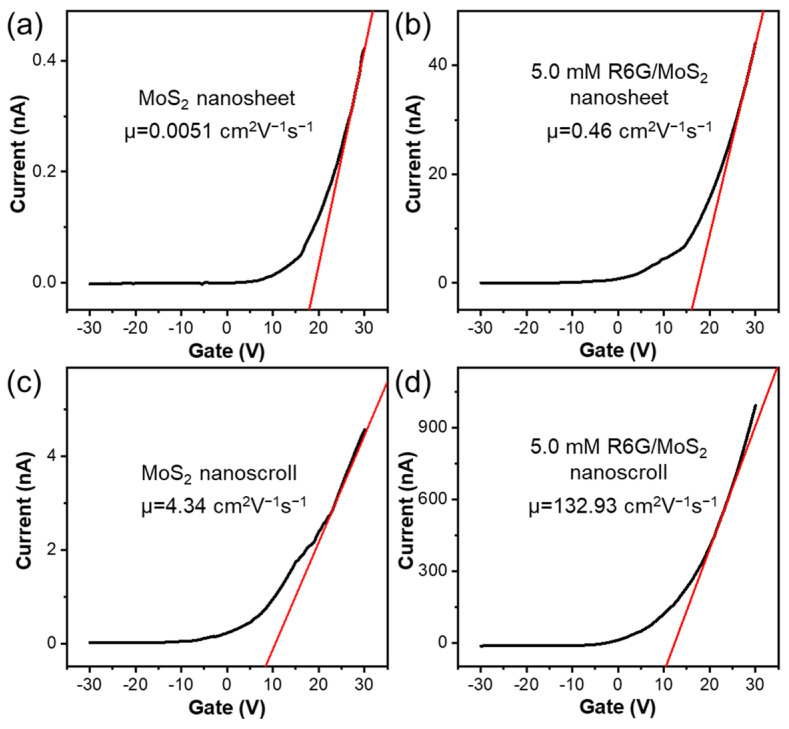
(**a**–**d**) The transfer curves of (**a**) MoS_2_ nanosheet, (**b**) R6G/MoS_2_ nanosheet, (**c**) MoS_2_ nanoscroll, and (**d**) R6G/MoS_2_ nanoscroll.

The optoelectronic performance of MoS_2_ nanosheet, R6G/MoS_2_ nanosheet, MoS_2_ nanoscroll, and R6G/MoS_2_ nanoscroll was investigated by measuring the carrier mobility, photoresponsivity, EQE, and detectivity of MoS_2_ nanosheet, R6G/MoS_2_ nanosheet, MoS_2_ nanoscroll, and R6G/MoS_2_ nanoscroll. The output curves and transfer characteristic curves of MoS_2_ nanosheet, R6G/MoS_2_ nanosheet, MoS_2_ nanoscroll, and R6G/MoS_2_ nanoscroll are shown in Figure 7 and Figure S6 in SI. All of these materials show n-type behavior over the bias range of −2 V to 2 V, with a voltage step of 0.8 V (Figure S6 in SI). The carrier mobility (µ) can be obtained from their transfer curves with the following equation,

(1) $$\mu =\frac{L}{w\times (\frac{\varepsilon_{0}\varepsilon_{r}}{d})\times V_{sd}}\frac{{dI}_{sd}}{{dV}_{g}}$$

where I_sd_/V_g_ is the slope of the linear region of the transmission curve, L is the length of the device channel, W is the width of the device channel, ℇ_0_ is 8.854 × 10^−12^ F/m, ℇ_r_ for SiO_2_ is 3.9, and d is the thickness of SiO_2_ (300 nm).

The carrier mobilities of MoS_2_ nanosheet, R6G/MoS_2_ nanosheet, and MoS_2_ nanoscroll are 0.0051, 0.46, and 4.34 cm^2^V^−1^s^−1^ (Figure 7a–c), respectively. While the carrier mobility of R6G/MoS_2_ nanoscroll is 132.93 cm^2^V^−1^s^−1^ (Figure 7d), which is four orders of magnitude higher than that of MoS_2_ nanosheet, indicating the great performance improvement from the scrolled structure in combination with the involvement of R6G. At the same time, the photoresponse time (τ_r_) and recovery time (τ_f_) of R6G/MoS_2_ nanoscroll are shorter than those of MoS_2_ nanosheet, R6G/MoS_2_ nanosheet, and MoS_2_ nanoscroll (Figure S7 in SI). The I_ds_-V_ds_ curves of MoS_2_ nanosheet, R6G/MoS_2_ nanosheet, MoS_2_ nanoscroll, and R6G/MoS_2_ nanoscroll-based devices were tested under 405 nm and 532 nm lasers (Figure S8 in SI). Their photocurrents increase with the increasing laser power. Compared with MoS_2_ nanosheet, the photocurrents of R6G/MoS_2_ nanosheet and R6G/MoS_2_ nanoscroll increase by at least two orders of magnitude. It has been demonstrated that the addition of R6G can effectively enhance the optoelectronic performance of these devices.

The photoresponsivity (R), external quantum efficiency (EQE), and detectivity (D*) are important parameters for evaluating the performance of the optoelectronic devices, which can be described by the following equations [31,32],

R = I_ph_/PS (2)

where I_ph_ is photocurrent, P is the laser power density, and S is the effective area of the device.

EQE = hcR/eλ(3)

where h is the Planck’s constant, c is the speed of light, e is the charge, and λ is the laser wavelength.

D* = R_λ_S^1/2^/(2eI_dark_)^1/2^ (4)

where R_λ_ is responsivity, S is the effective irradiated area, e is the charge, and I_dark_ is dark current.

**Figure 8:**
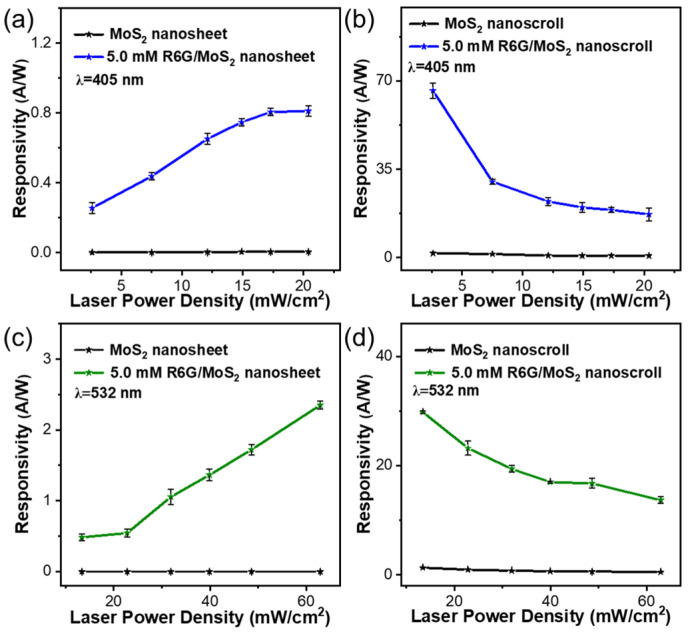
The photoresponsivities of (**a**,**c**) MoS_2_ and R6G/MoS_2_ nanosheets as well as (**b**,**d**) MoS_2_ and R6G/MoS_2_ nanoscrolls under (**a**,**b**) 405 nm and (**c**,**d**) 532 nm lasers as a function of laser power density.

We first explored the photoresponsivity of MoS_2_ nanosheets modified by R6G solutions with concentrations ranging from 0 mM to 7.0 mM. The photoresponsivity increased with the increase in laser power and R6G concentration, as shown in Figure S9 in SI. The photoresponsivities of MoS_2_ and R6G/MoS_2_ nanosheet-based devices at 405 nm and 532 nm increase with increasing laser power density, and this phenomenon is also observed in WS_2_ and R6G/WS_2_ nanosheet-based devices (Figure S10 in SI). This is attributed to the fact that under laser irradiation, more electron-hole pairs are generated and then transported with the contribution of R6G, resulting in higher photocurrents. Figure 8 demonstrates the photoresponsivity of MoS_2_ nanosheet, R6G/MoS_2_ nanosheet, MoS_2_ nanoscroll, and R6G/MoS_2_ nanoscroll-based devices under 405 nm and 532 nm lasers, respectively. The MoS_2_ nanosheets show photoresponsivities of 6.70 × 10^−3^ A/W and 6.63 × 10^−4^ A/W (Table 1), respectively. While the photoresponsivities of R6G/MoS_2_ nanosheets are improved by 2-3 orders of magnitude with the addition of R6G (0.81 A/W and 2.35 A/W) (Table 1, Figure 8a,c). The photoresponsivities of R6G/MoS_2_ nanoscrolls were also investigated under 405 and 532 nm lasers with various laser power densities, as shown in Figure S11 in SI. The photoresponsivities of MoS_2_ nanoscrolls and R6G/MoS_2_ nanoscroll-based devices decrease with increasing laser power density, and similar phenomena also exist in WS_2_ and R6G/WS_2_ nanoscroll-based devices (Figure S12 in SI). Surprisingly, the photoresponsivities of 5.0 mM R6G/MoS_2_ nanoscrolls increased to 66.07 A/W and 29.80 A/W, which are improved by four orders of magnitude compared to MoS_2_ nanosheets (Figure 8b,d and Table 1). Compared to other photodetectors, it shows excellent photoelectric performance (Table S1 in SI). The external quantum efficiency and detectivity were also improved by four and one orders of magnitude under the same conditions (Table 1), implying the important role of R6G in enhancing the optoelectronic performance. The maximum power density of a 532 nm laser is about 3 times higher than that of a 405 nm laser. Due to the limited light absorption of the monolayer MoS_2_ nanosheet, the R6G with high light absorption plays an important role in enhancing the optoelectronic performance of the R6G/MoS_2_ nanosheet. As shown in Figure S8 in SI, the photocurrent of the R6G/MoS_2_ nanosheet under 532 nm laser irradiation is around 9 times higher than that under 405 nm laser irradiation. In this case, the ratio of photocurrent to power density of the R6G/MoS_2_ nanosheet is around 3 times higher under a 532 nm laser than that under a 405 nm laser. Therefore, the R6G/MoS_2_ nanosheet shows higher responsivity and EQE under a 532 nm laser. While for the R6G/MoS_2_ nanoscroll, both the scrolled structure and the encapsulation of R6G play an important role in enhancing the optoelectronic performance. The photocurrent of the R6G/MoS_2_ nanoscroll under a 532 nm laser is no more than 3 times higher than that under a 405 nm laser. Meanwhile, the minimum power density of a 532 nm laser is around 5 times higher than that of a 405 nm laser. Thus, the ratio of photocurrent to power density of the R6G/MoS_2_ nanoscroll under a 532 nm laser is less than that under a 405 nm laser. As a consequence, the responsivity and EQE of the R6G/MoS_2_ nanoscroll under a 405 nm laser are higher than those under a 532 nm laser.

The photoresponsivities of R6G/MoS_2_ nanoscrolls show a different trend from those of R6G/MoS_2_ nanosheets under increasing laser power density, which could be explained as follows. The organic photosensitive material R6G has a large absorption coefficient, which can enhance the absorption of light. When it is combined with MoS_2_ nanosheets to form the R6G/MoS_2_ nanosheets, a type-II heterojunction interface is presented [31], where electrons tend to flow from R6G to MoS_2_, and holes tend to flow from MoS_2_ to R6G. Thus, the electrons and holes can be separated efficiently with the addition of R6G, and the photoresponsivity can be enhanced (Figure S13 in SI). Similar phenomena have also been reported in silicon photodetectors [41], planar metal-insulator-semiconductor-insulator-metal diodes [42], and MoS_2_/AsP van der Waals heterostructure diodes [43]. The R6G/MoS_2_ nanosheets have only one type-II hetero-interface, while multiple heterojunction interfaces are formed in the R6G/MoS_2_ nanoscrolls. These multiple interfaces could exhibit much higher light absorption efficiency and promote the generation and separation of photogenerated electron-hole pairs. Therefore, a large number of electron-hole pairs are generated in a short time under laser irradiation, and thus the photocurrent is saturated subsequently (Figure S13 in SI). Therefore, the increased laser power resulted in decreased photoresponsivity of R6G/MoS_2_ nanoscrolls.

In order to show the stability of R6G encapsulated in the MoS_2_ nanoscroll, we have measured the optoelectronic performance of the R6G/MoS_2_ nanosheet and nanoscroll after being stored in ambient conditions for 6 months. As shown in Figure S14 in SI, the photocurrent and responsivity of the R6G/MoS_2_ nanosheet greatly decreased after 6 months. While the R6G/MoS_2_ nanoscroll still shows comparable photocurrent and responsivity even after 6 months, indicating the advantage of encapsulation in nanoscroll. Encapsulating R6G in MoS_2_ nanoscroll can prevent the influence of oxygen and water on the degradation of R6G, which can maintain the optoelectronic performance of R6G/MoS_2_ nanoscroll. However, the exposure of R6G to oxygen and water can degrade R6G, and thus decrease its optoelectronic performance. In addition, the intensity of Raman peaks of R6G in the R6G/MoS_2_ nanosheet and R6G/MoS_2_ nanoscroll also showed a similar trend. Integration of optical antennas [44], dielectric engineering [45], and metalens [46], as well as embedding charge puddles [47], with TMDC nanosheets, have also been reported to show excellent optoelectronic performance. In our work, encapsulating R6G in MoS_2_ nanoscrolls not only enhances the optoelectronic performance but also prevents the degradation of R6G in ambient conditions, as shown in Figure S14.

## 3. Materials and Methods

### 3.1. Preparation of MoS_2_ and R6G/MoS_2_ Nanosheets

MoS_2_ was grown by the CVD method. Firstly, 0.3 g S (Macklin Biochemical Co., Ltd., Shanghai, China) and 3.0 mg MoO_3_ (Energy Chemical, Shanghai, China) powders were placed in two porcelain boats. Secondly, a 300 nm SiO_2_/Si substrate was placed on the porcelain boat with MoO_3_ powder, and then the porcelain boat was placed in the center of the tube furnace. The porcelain boat containing sulfur powder was placed in the upstream area. The distance between the two porcelain boats was 16.5 cm, and they were kept at 670 °C for 5 min. Finally, the monolayer MoS_2_ nanosheets were successfully obtained.

For the preparation of R6G/MoS_2_ nanosheets, the first step was to dissolve R6G in ethanol solutions with concentrations of 0.5, 1.0, 2.0, 5.0, and 7.0 mM. After that, the CVD-grown monolayer MoS_2_ nanosheets were immersed into the R6G/ethanol solution for a certain time. After that, the excess R6G solution was rinsed off with deionized water, and then the samples were dried with nitrogen. Thus, clean and uniform R6G/MoS_2_ nanosheets were obtained (Figure S15 in SI).

### 3.2. Preparation of MoS_2_ Nanoscrolls and R6G/MoS_2_ Nanoscrolls

Firstly, ammonia and ethanol were mixed in volume ratios of 1:1, 3:1, and 5:1. The MoS_2_ and R6G/MoS_2_ nanosheets were heated at 100 °C for 2 min. After that, 10 μL of ammonia/ethanol mixed solutions was deposited dropwise onto the nanosheets to explore the formation of nanoscrolls. Subsequently, the ammonia/ethanol mixed solution with a volume ratio of 3:1 was optimized to prepare MoS_2_ and R6G/MoS_2_ nanoscrolls. It was found that the nanosheets were transformed into nanoscrolls in a short time under this condition. Thus, large-area and high-quality MoS_2_ and R6G/MoS_2_ nanoscrolls were obtained (Figure S15 in SI).

### 3.3. Characterization of MoS_2_ and R6G/MoS_2_ Nanosheets and Nanoscrolls

Firstly, optical microscopy (Axio Scope A1, Zeiss, Oberkochen, Germany) was used to observe the change of MoS_2_ nanosheets and nanoscrolls before and after the modification of R6G. After that, the as-obtained R6G/MoS_2_ nanosheets and nanoscrolls were characterized by an atomic force microscope (Dimension ICON with Nanoscope V controller, Bruker, Billerica, MA, USA) to observe the height change of the sample before and after the modification of R6G. A Raman spectrometer (HR Evolution, Horiba Jobin Yvon, Paris, France) with a 532 nm laser was used to collect the Raman spectra of the MoS_2_, R6G/MoS_2_ nanosheets, and nanoscrolls. Transmission electron microscopy (JEOL JEM-2100, Tokyo, Japan) was used to characterize the distribution of Mo, S, and Cl elements in MoS_2_, R6G/MoS_2_ nanosheets and nanoscrolls.

### 3.4. Device Fabrication and Measurement

Cr film with a thickness of 5.0 nm was first deposited on the surface of the material as a bonding layer between the material and the Au electrode. Then, 50 nm of Au film was deposited on the surface of R6G/MoS_2_ nanosheets and nanoscrolls as a metallic contact by using a 300-mesh copper mesh as a mask in a thermal evaporator. The obtained device is characterized on a semiconductor parameter analyzer (Keithley 4200, Beaverton, OR, USA). Nanosheets and nanoscroll-based devices were irradiated by 405 nm and 532 nm lasers with various laser power densities to obtain photocurrents, response times, recovery times, and transfer curves. The responsivity, EQE, and mobility were calculated based on the effective light–absorbing area (Figure S16 in SI) of the nanosheets and nanoscrolls. The response time (τ_r_) represents the duration for photocurrent ascending from 10% to 90% of the pulse peak, while the decay time (τ_f_) represents the duration for photocurrent descending from 90% to 10% of the pulse peak. In this process, the time-resolved photoresponse was measured by switching the laser on and off at V_ds_ = 10^−5^ V.

## 4. Conclusions

In conclusion, we have successfully obtained R6G/MoS_2_ nanosheets by immersing CVD-grown monolayer MoS_2_ nanosheets in an R6G solution. Large-scale R6G/MoS_2_ nanoscrolls with lengths of hundreds of micrometers were then obtained in the presence of a mixed solution of ethanol and ammonia. Various characterization instruments, such as optical microscopy, atomic force microscopy, and Raman spectroscopy, showed that R6G had been successfully deposited on the MoS_2_ nanosheets and encapsulated in the MoS_2_ nanoscrolls. Under 405 nm and 532 nm laser irradiation, the photoresponsivities of R6G/MoS_2_ nanoscrolls increased by four orders of magnitude compared to those of the MoS_2_ nanosheets, indicating that the organic dye R6G plays an important role in improving the optoelectronic performance of the MoS_2_ nanosheets and nanoscrolls. A type-II heterojunction forms between the interface of R6G and MoS_2_ nanosheets, which allows electrons to be rapidly transferred from R6G to MoS_2_, resulting in higher photocurrent, mobility, and photoresponsivity. The R6G/MoS_2_ nanoscrolls have multiple type-II heterojunction interfaces, which greatly improves the light absorption ability and facilitates effective electron–hole separation. The 1D structure of R6G/MoS_2_ nanoscrolls also constrains electron transportation along the long-axis direction, so that the photocurrent is much higher than that of R6G/MoS_2_ nanosheets. Therefore, R6G/MoS_2_ nanoscrolls could be considered a candidate material for high-performance optoelectronic devices in the future.

## Figures and Tables

**Table 1:** The photoresponsivity (R), external quantum efficiency (EQE), detectivity (D*), and mobility (μ) of MoS_2_ nanosheet, R6G/MoS_2_ nanosheet, MoS_2_ nanoscroll, and R6G/MoS_2_ nanoscroll under 405 nm and 532 nm lasers.

Device	R (A/W) 405 nm	R (A/W) 532 nm	EQE (%) 405 nm	EQE (%) 532 nm	D* (cm·Hz^1/2^W^−1^) 405 nm	D* (cm·Hz^1/2^W^−1^) 532 nm	μ (cm^2^V^−1^s^−1^)
MoS_2_ nanosheets	6.70 × 10^−3^	6.63 × 10^−4^	2.1	0.16	7.5 × 10^10^	3.49 × 10^9^	0.0051
5.0 mM R6G/MoS_2_ nanosheets	0.81	2.35	249	550	1.07 × 10^12^	2.65 × 10^11^	0.46
MoS_2_ nanoscrolls	1.71	1.32	524.97	98.28	4.36 × 10^10^	1.75 × 10^10^	4.34
5.0 mM R6G/MoS_2_ nanoscrolls	66.07	29.80	20,261	6957	1.25 × 10^12^	9.73 × 10^10^	132.93

## Data Availability

The original contributions presented in the study are included in the article/Supplementary Material, further inquiries can be directed to the corresponding authors.

## Supplementary Materials

The supporting information is available free of charge at https://www.mdpi.com/article/10.3390/molecules29122799/s1, The optical microscopy (OM) images of R6G/MoS_2_ nanoscrolls prepared using NaHCO_3_ solution, ethanol and water; OM images of R6G/MoS_2_ nanoscrolls prepared by ammonia/ethanol mixed solutions with volume ratios of 1:1, 3:1 and 5:1; the atomic force microscope (AFM) images of R6G/MoS_2_ nanosheets with the immersion time at 1 min, 3 min, 5 min, 7 min, 10 min, 30 min, and 60 min; HR-TEM image of R6G/MoS_2_ nanoscrolls and the energy dispersive spectroscopy analysis on the distribution of S, Mo, and Cl elements; the output curves of MoS_2_, 5.0 mM R6G/MoS_2_ nanosheets and nanoscrolls with gate voltage ranging from −2 V to 2 V at a step of 0.8 V; the light response time, recovery time of MoS_2_ nanosheets, 5.0 mM R6G/MoS_2_ nanosheets, MoS_2_ nanoscrolls and 5.0 mM R6G/MoS_2_ nanoscrolls under 405 nm, 532 nm lasers; the I_ds_-V_ds_ curves of MoS_2_ nanosheets, 5.0 mM R6G/MoS_2_ nanosheets, MoS_2_ nanoscrolls and 5.0 mM R6G/MoS_2_ nanoscrolls under 405 nm, 532 nm lasers; the photocurrent and photoresponsivity of R6G/MoS_2_ and R6G/WS_2_ nanosheets as well as nanoscrolls as a function of laser power density under 405 nm and 532 nm lasers; mechanistic explanation of optoelectronic performance enhancement. References [20,48,49,50,51] are cited in the supplementary materials.
