# Supplementary material for: Rhodamine 6G/Transition Metal Dichalcogenide Hybrid Nanoscrolls for Enhanced Optoelectronic Performance
Source: Molecules. 2024 Jun 12;29(12):2799. doi: 10.3390/molecules29122799 (PMC11207076; doi:10.3390/molecules29122799)
Supplement: Supplementary file 1 [file molecules-29-02799-s001.zip › molecules-3035329-supplementary.pdf]

Supporting Information

Rhodamine 6G/Transition Metal Dichalcogenide Hybrid Nanoscrolls for Enhanced Optoelectronic Performance

Huihui Ye, Hailun Tang, Shilong Yu, Yang Yang and Hai Li *

Key Laboratory of Flexible Electronics (KLOFE), Institute of Advanced Materials (IAM), Nanjing Tech University (NanjingTech), Nanjing 211816, China
* Correspondence: iamhli@njtech.edu.cn

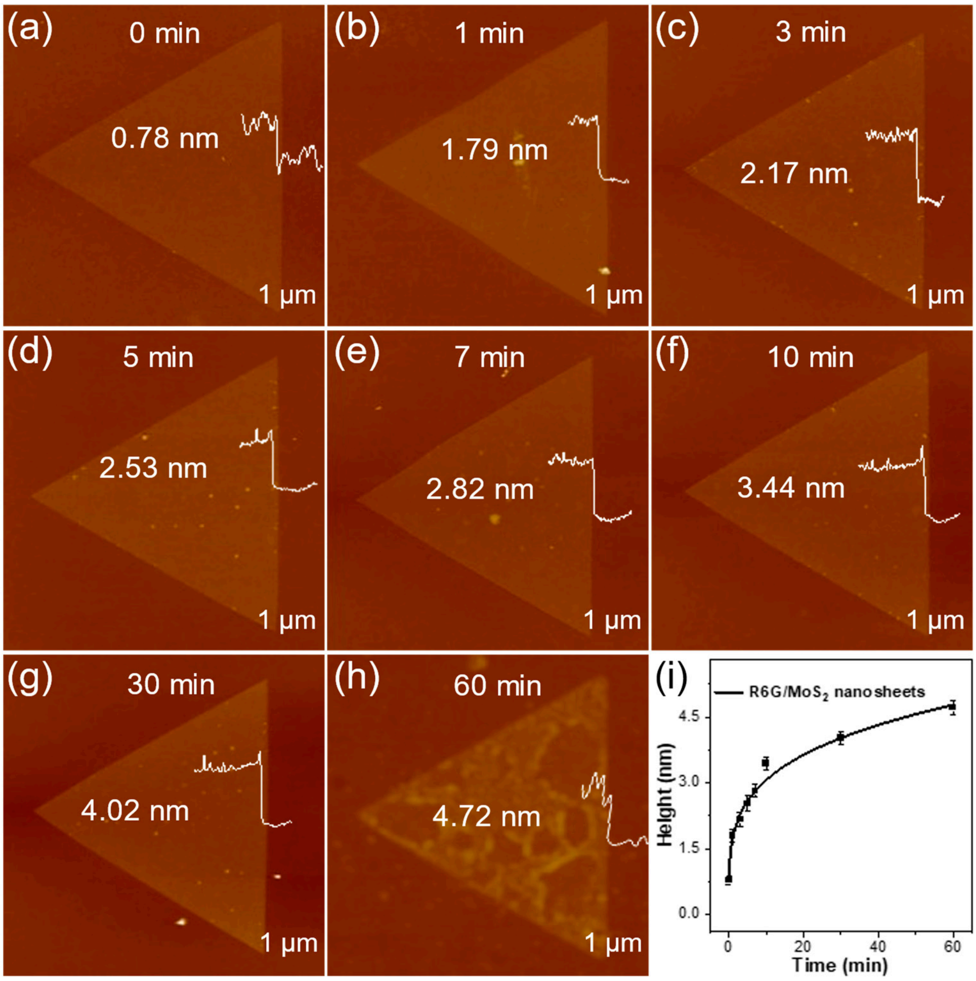

Figure S1. The AFM height images of R6G/MoS_2 nanosheets immersed into 5.0 mM R6G for (a) 0 min, (b) 1 min, (c) 3 min, (d) 5 min, (e) 7 min, (f) 10 min, (g) 30 min, and (h) 60 min. (i) The thickness of R6G/MoS_2 nanosheets as function of concenteation of R6G solition.

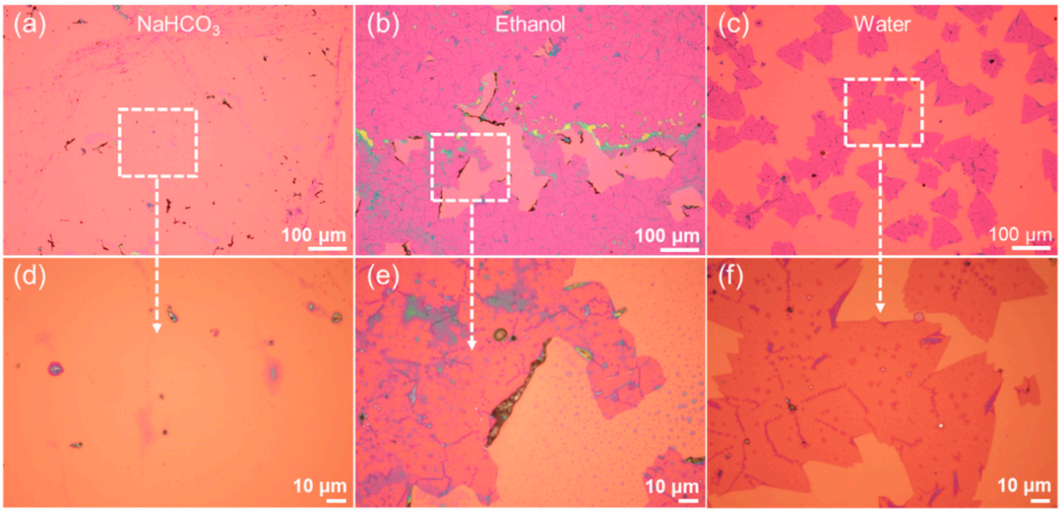

Figure S2. (a-c) The OM images of R6G/MoS_2 nanoscrolls prepared by using (a) $NaHCO_3$ solution, (b) ethanol and (c) water. (d-f) The corresponding magnified images of white dashed boxes shown in (a-c).

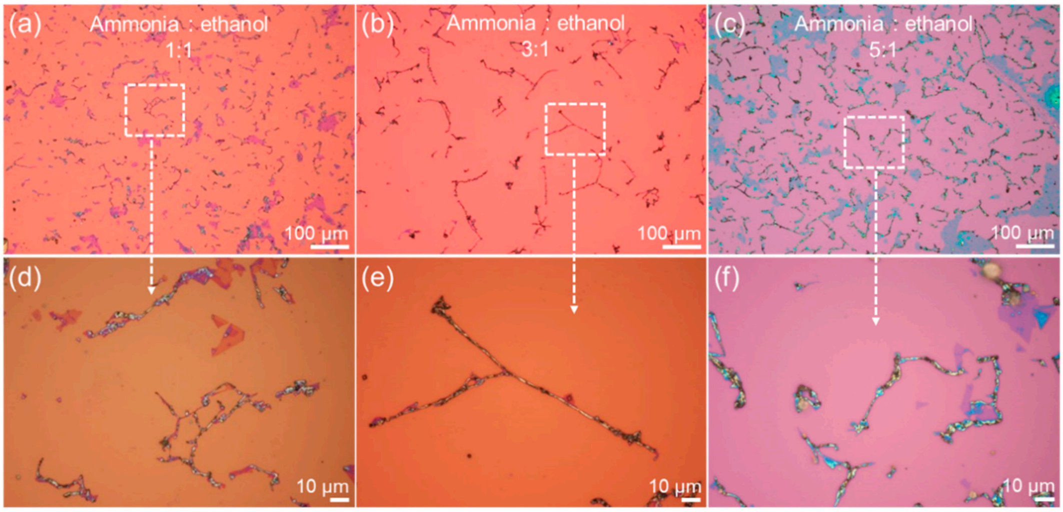

Figure S3. (a-c) The OM images of R6G/MoS_2 nanoscrolls prepared using ammonia/ethanol mixed solutions with volume ratios of (a) 1:1, (b) 3:1, and (c) 5:1. (d-f) The corresponding magnified images of white dashed boxes shown in (a-c).

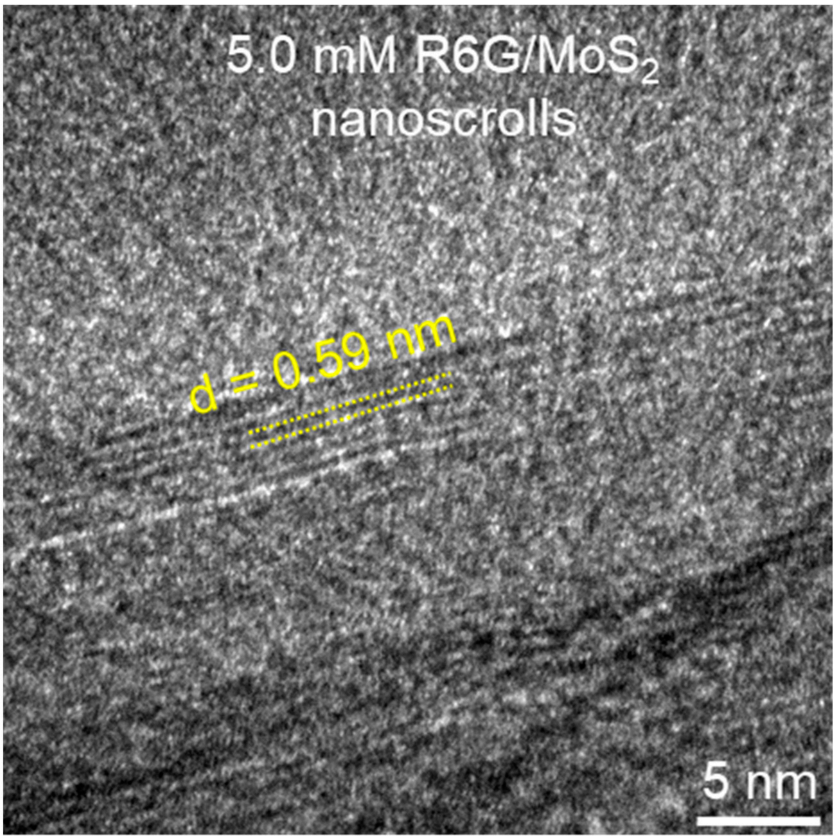

Figure S4. HR-TEM image of R6G/MoS_2 nanoscroll.

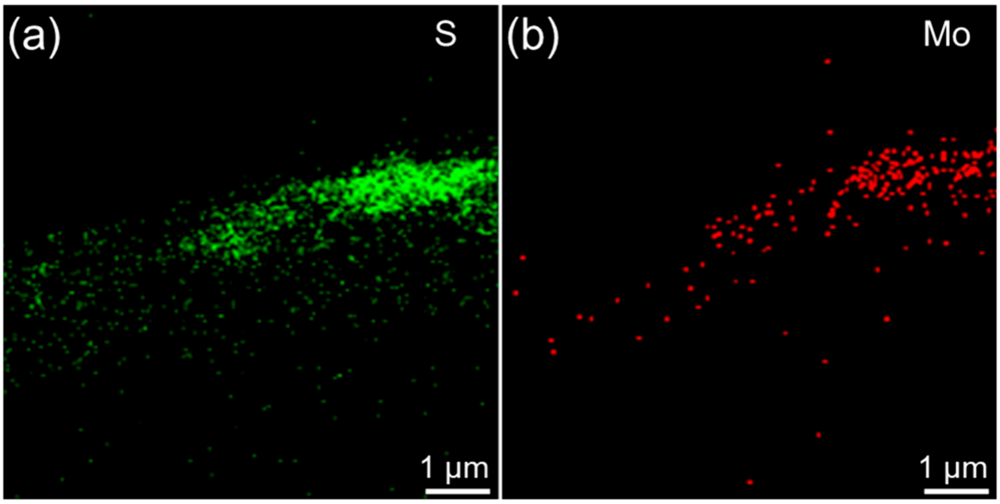

Figure S5. The energy dispersive spectroscopy mapping analysis on the distribution of (a) S and (b) Mo elements in the R6G/MoS_2 nanoscroll.

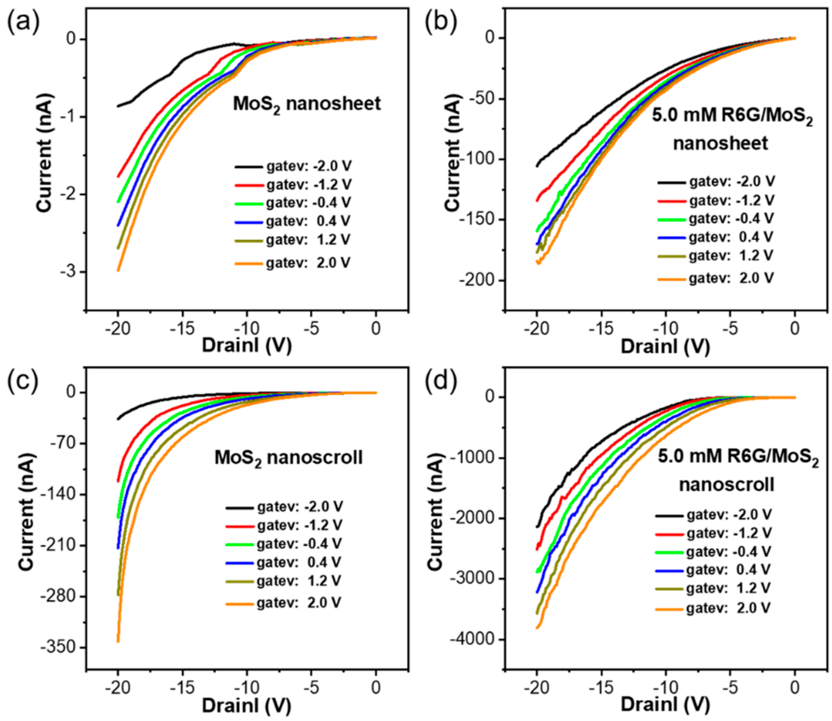

Figure S6. The output curves of (a) MoS_2 nanosheet, (b) R6G/MoS_2 nanosheet, (c) MoS_2 nanoscroll, and (d) R6G/MoS_2 nanoscroll with gate voltage ranging from−2 V to 2 V at a step of 0.8 V.

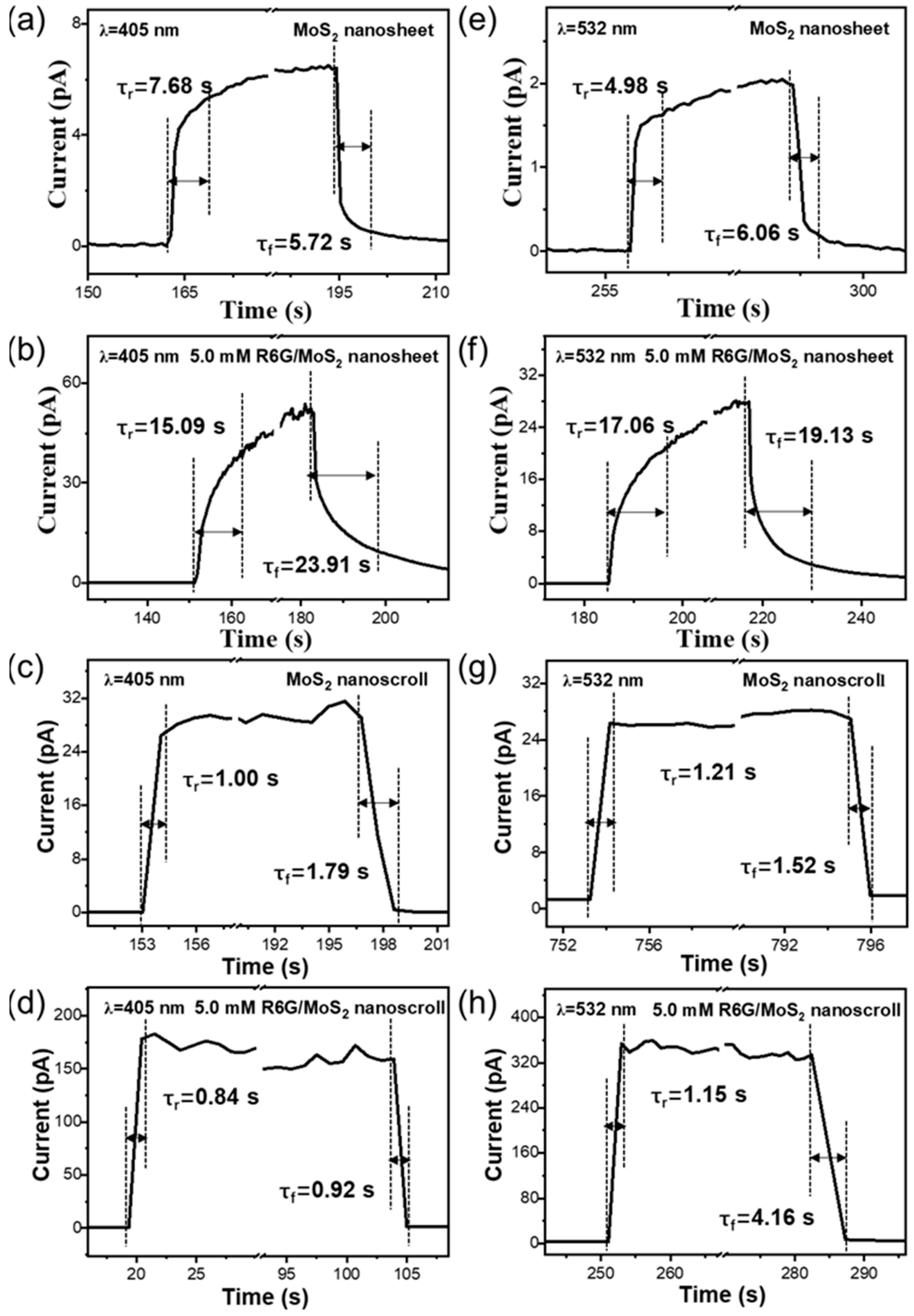

Figure S7. The response time and recovery time of (a, e) MoS_2 nanosheet, (b, f) 5.0 mM R6G/MoS_2 nanosheet, (c, g) MoS_2 nanoscroll and (d, h) 5.0 mM R6G/MoS_2 nanoscroll under (a-d) 405 nm and (g-h) 532 nm lasers.

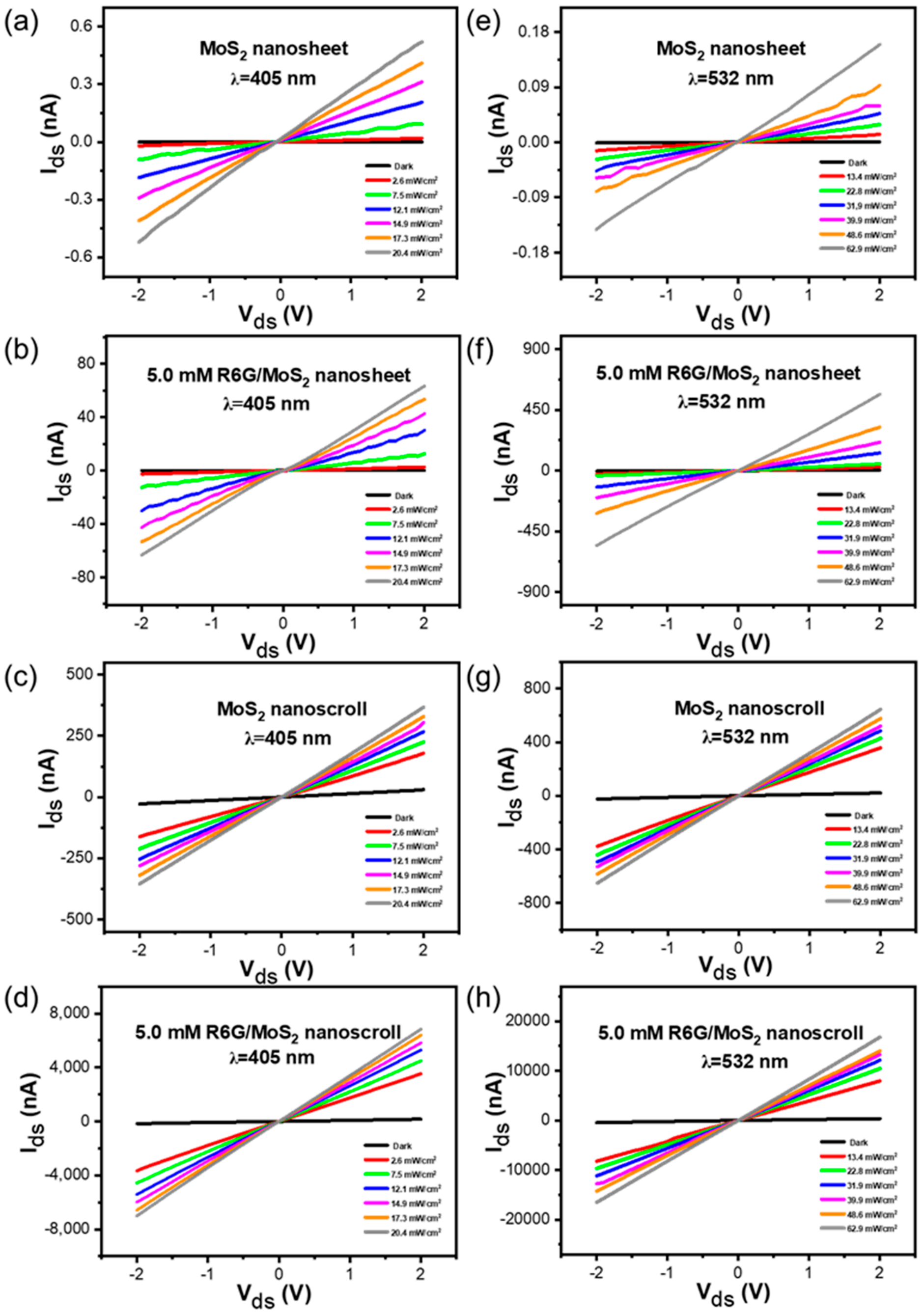

Figure S8. The I_{ds}-V_{ds} curves of (a, e) MoS_2 nanosheet, (b, f) 5.0 mM R6G/MoS_2 nanosheet, (c, g) MoS_2 nanoscroll and (d, h) 5.0 mM R6G/MoS_2 nanoscroll under (a-d) 405 nm and (e-h) 532 nm lasers.

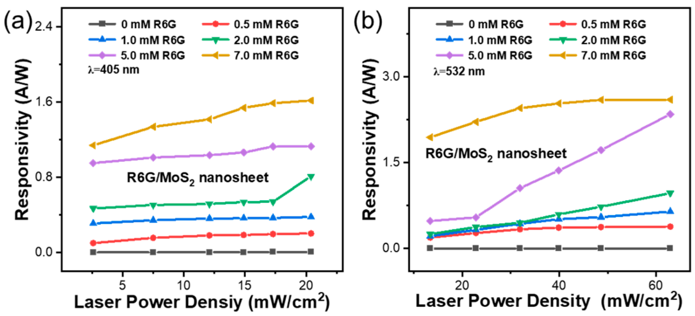

Figure S9. The photoresponsivities of R6G/MoS_2 nanosheets as a function of laser power density under (a) 405 nm and (b) 532 nm lasers.

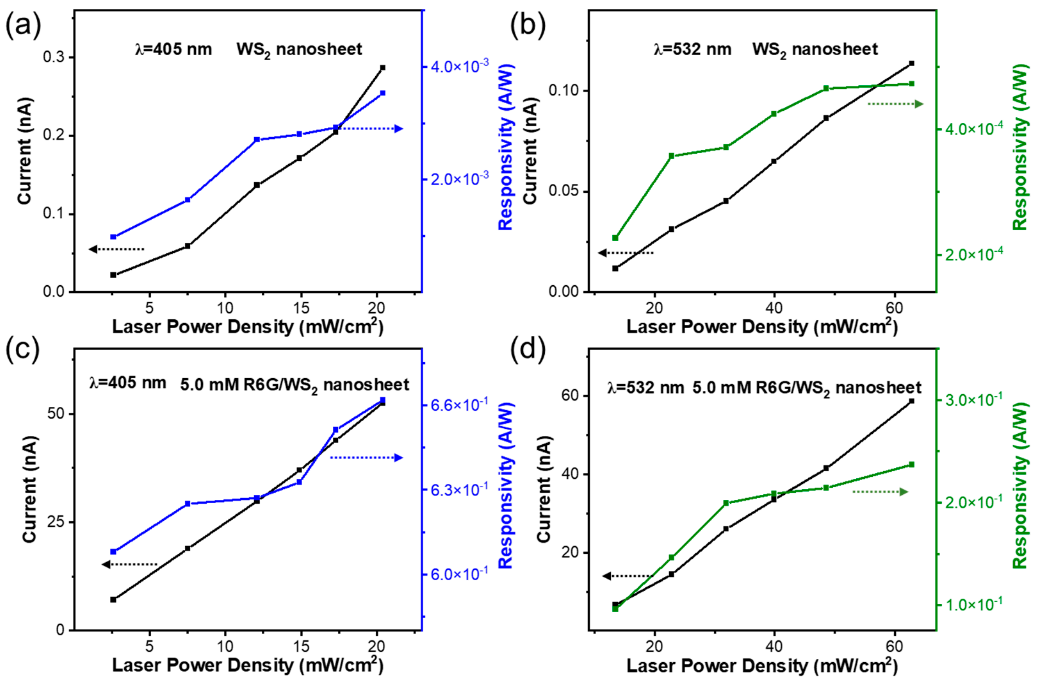

Figure S10. The photocurrent and photoresponsivities of (a, b) WS_2 nanosheet and (c, d) 5.0 mM R6G/WS_2 nanosheet as a function of laser power density under (a, c) 405 nm and (b, d) 532 nm lasers.

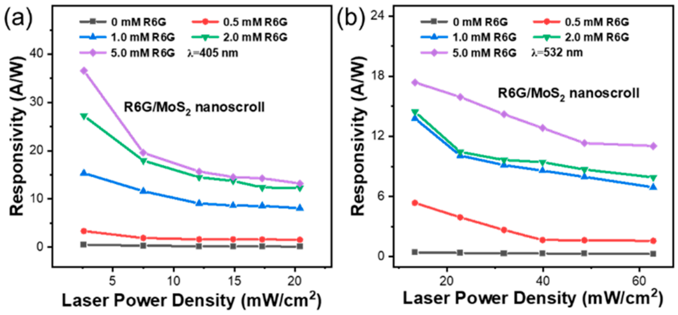

Figure S11. The photoresponsivities of R6G/MoS_2 nanoscrolls as a function of laser power density under (a) 405 nm and (b) 532 nm lasers.

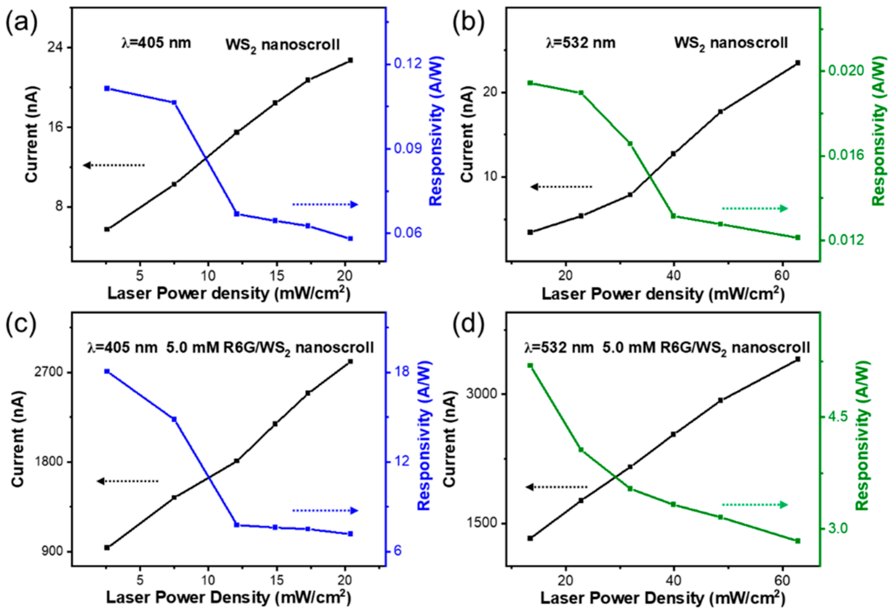

Figure S12. The photocurrent and photoresponsivities of (a, b) WS_2 nanoscroll and (c, d) 5.0 mM R6G/WS_2 nanoscroll as a function of laser power density under (a, c) 405 nm and (b, d) 532 nm lasers.

Table S1. Performance comparison of this work to the lately reported TMDC photodetector.

Material	R (A/W)	EQE (%)	D* (cm·Hz$^{1/2}$ W^{-1})	Reference
R6G/MoS_2 nanoscroll	66.07	20261	1.25×10^{12}	this work
MoS_2 nanotubes	20.6	4947.6	1.94×10^{12}	ACS Appl. Mater. Interfaces 2024 DOI: 10.1021/acsami.4c01823
In_2Se_3-WS_2 heterojunction	6.35		3.1×10^{11}	Opt. Mater 2024, 149, 115052
Zn-MoS_2 heterojunction	1.45	30.2	1.28×10^{12}	J. Mater. Chem. C 2024, 12, 5247-5256
Te/2D $MoSe_2$	0.328	79	8.2×10^{9}	Adv. Funct 2024, 34, 2311134
BP/R6G/MoS_2 heterojunction	0.19	44.9	6.1×10^{10}	Nano Res. 2023, 16, 10537

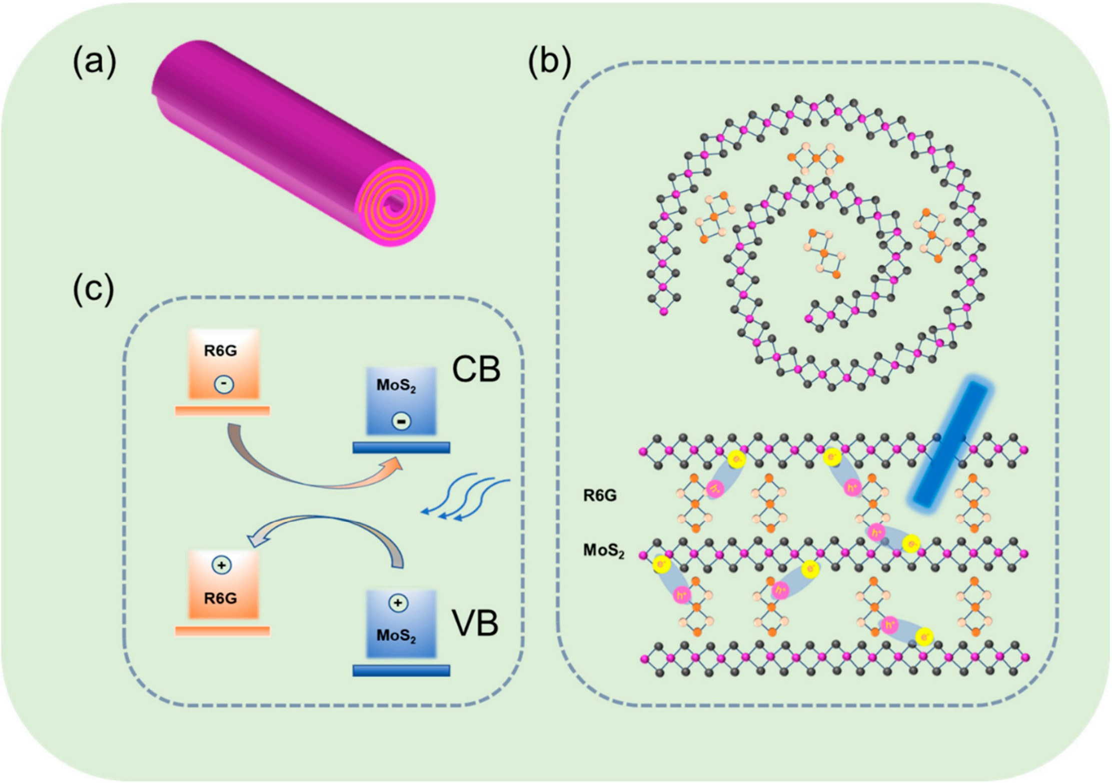

Figure S13. (a) The scheme of R6G/MoS_2 nanosscroll. (b) Interlayer transport behaviour of photogenerated carriers in multiple interfaces of R6G/MoS_2 nanoscroll. (c) The schematic diagram of energy-band arrangement of R6G/MoS_2 nanoscroll.

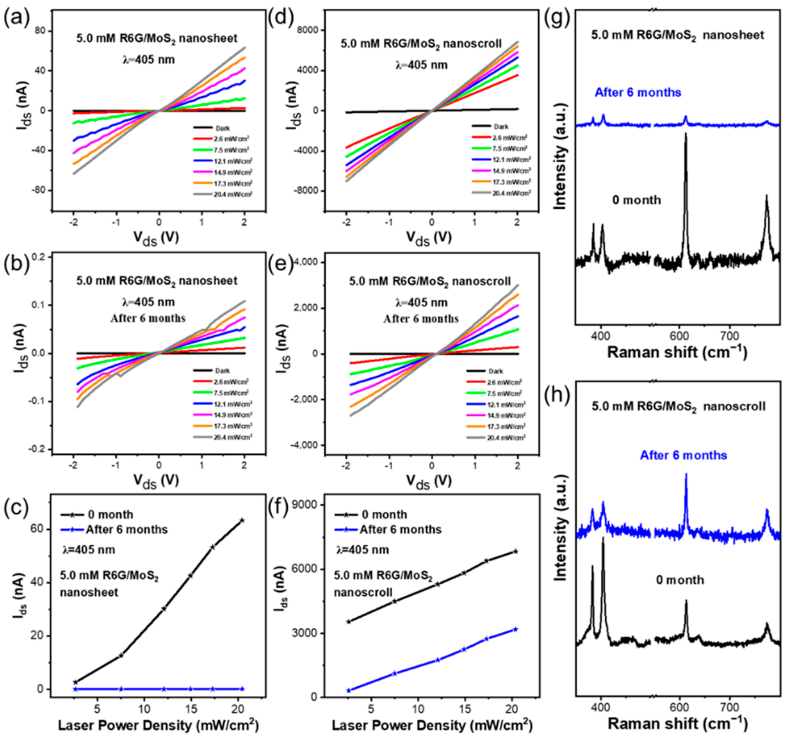

Figure S14. The photocurrent plots of 5.0 mM R6G/MoS_2 nanosheet and nanoscroll measured (a, d) before and (b, e) after being stored in ambient conditions for 6 months. (c, f) The photocurrent plots and (g-h) Raman spectra of 5.0 mM R6G/MoS_2 nanosheet and nanoscroll before and after being stored in ambient conditions for 6 months.

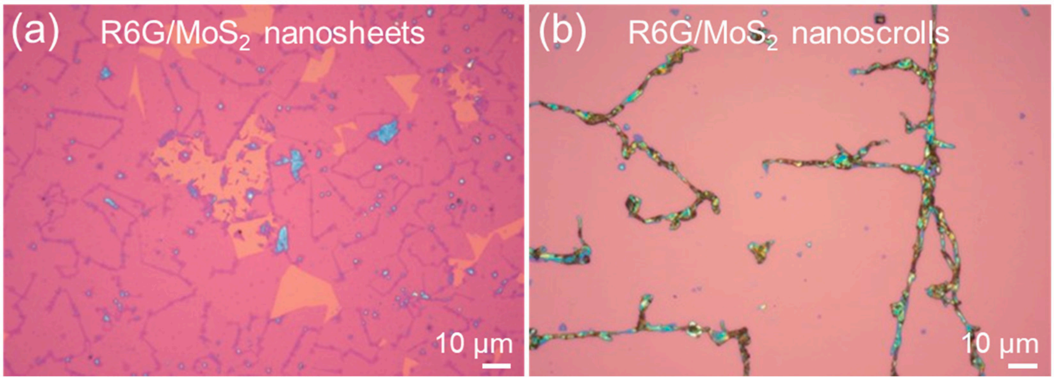

Figure S15. The OM images of (a) R6G/MoS_2 nanosheets and (b) R6G/MoS_2 nanoscrolls.

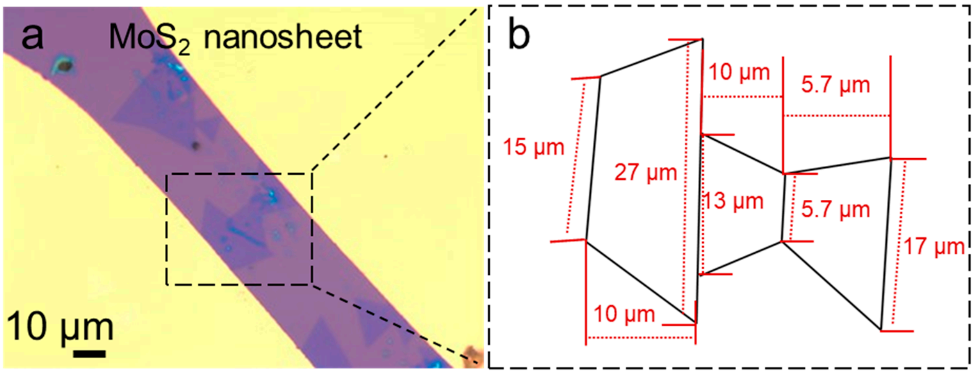

Figure S16. The (a) OM image and (b) corresponding schematic illustration of MoS_2 nanosheet based device.
